# An Objective Injury Threshold for the Maximum Principal Strain Criterion for Brain Tissue in the Finite Element Head Model and Its Application

**DOI:** 10.3390/bioengineering11090918

**Published:** 2024-09-13

**Authors:** Yuting Zhang, Liqun Tang, Yiping Liu, Bao Yang, Zhenyu Jiang, Zejia Liu, Licheng Zhou

**Affiliations:** Department of Engineering Mechanics, School of Civil Engineering and Transportation, South China University of Technology, No. 381, Wushan Road, Guangzhou 510000, China; ctzhangyuting@mail.scut.edu.cn (Y.Z.); tcypliu@scut.edu.cn (Y.L.); zhenyujiang@scut.edu.cn (Z.J.); zjliu@scut.edu.cn (Z.L.); ctlczhou@scut.edu.cn (L.Z.)

**Keywords:** brain injury thresholds, FEHM, maximum principal strain, supra-tentorium cerebelli, visual memory impairment

## Abstract

Although the finite element head model (FEHM) has been widely utilized to analyze injury locations and patterns in traumatic brain injury, significant controversy persists regarding the selection of a mechanical injury variable and its corresponding threshold. This paper aims to determine an objective injury threshold for maximum principal strain (MPS) through a novel data-driven method, and to validate and apply it. We extract the peak responses from all elements across 100 head impact simulations to form a dataset, and then determine the objective injury threshold by analyzing the relationship between the combined injury degree and the threshold according to the stationary value principle. Using an occipital impact case from a clinical report as an example, we evaluate the accuracy of the injury prediction based on the new threshold. The results show that the injury area predicted by finite element analysis closely matches the main injury area observed in CT images, without the issue of over- or underestimating the injury due to an unreasonable threshold. Furthermore, by applying this threshold to the finite element analysis of designed occipital impacts, we observe, for the first time, supra-tentorium cerebelli injury, which is related to visual memory impairment. This discovery may indicate the biomechanical mechanism of visual memory impairment after occipital impacts reported in clinical cases.

## 1. Introduction

Biomechanical analysis of traumatic brain injury (TBI) plays an essential role in revealing the injury mechanism [1,2,3] and advancing injury prevention [4,5,6]. This is due to its ability to establish correlations and provide a deeper understanding of the relationship between mechanical injury and clinical consequences [7]. Over the past half century, research on finite element head models (FEHMs) in predicting brain injury locations and patterns has led to a consensus that primary brain injuries are often accompanied by high strain or stress responses [8]. Various injury criteria have been proposed at the kinematic [9,10,11,12], tissue [13,14,15,16,17], and axonal levels [18,19,20,21], with the maximum principal strain (MPS) being one of the most widely used tissue-level injury criteria [22]. Ghajari et al. demonstrated the biomechanical mechanisms behind chronic traumatic encephalopathy (CTE) pathology, using an MPS threshold of 0.4 for permanent brain injury [23].

When the head is subjected to external impacts, injuries can range from brain dysfunction to skull fractures. Recently, Wan et al. proposed a 3D hierarchical fully convolutional network (FCN) to predict the exact location of defective structures [24], providing a pathway for the precise identification of physical injuries such as skull fractures. However, image-based deep learning methods like this are less suitable for diagnosing brain injuries, especially in cases of mild traumatic brain injury (mTBI). For the biomechanical assessment of brain injuries, the standard approach typically involves the finite element reconstruction of clinical cases, combined with logistic regression analysis to determine injury thresholds [14]. The most accurate injury risk curves are constructed by correlating the global maximum responses in regions of interest with clinically determined injury statuses. From these curves, an injury threshold associated with a given risk level is established. Kleiven et al. analyzed 58 head impact cases and concluded that when the MPS in the corpus callosum or gray matter reached 0.21 or 0.26, respectively, there was a 50% risk of concussion [25]. Patton et al., after analyzing 40 additional head impacts, suggested injury thresholds for a 50% concussion risk to be 0.13, 0.15, and 0.26 for the thalamus, corpus callosum, and white matter regions, respectively [17]. Subsequently, Clark et al. proposed that the injury threshold for the entire brain should be 0.24 [26]. Notably, the corpus callosum is recognized as the primary injury region for concussions [16,27,28] and Diffuse Axonal Injury (DAI) [29,30], but there remains up to a 40% deviation in recommended injury thresholds derived from risk curves. The “clinical case-based” method identified earlier presents two significant limitations: (1) a relative scarcity of uninjured cases compared to injured ones [8,17]; and (2) the use of the global maximum response, which inadequately characterizes injury status and can lead to situations where similar responses correspond to opposite injury outcomes. Moreover, this method has also been applied to injury criteria based on variables such as Von Mises stress [14,31], shear stress [15], and axonal strain [20,32]. Thus, there is a clear need for the development of more appropriate methods for determining tissue-level injury thresholds.

The validation of newly proposed injury thresholds is crucial, particularly in aligning them with clinical outcomes. Viano et al. were the first to establish a correlation between the physical forces occurring during concussions and the clinical characteristics observed in actual injured athletes [27]. They demonstrated a significant link between high strain or strain rate in brain regions such as the midbrain, hippocampus, and corpus callosum, and clinical symptoms like cognitive/memory problems and loss of consciousness. Similarly, Kleiven et al. reconstructed a motorcycle crash and successfully predicted, in part, the hematoma location visible in the CT images using MPS injury thresholds [25]. More recently, Yuan et al. reconstructed a skiing accident and found that the regions of brain tissue exhibiting high strain levels closely corresponded to areas of edema and micro-bleeding seen in medical images [33]. It is noteworthy, however, that these high strain levels reached approximately 0.65, while areas with strain levels below this threshold have not been thoroughly examined in relation to clinical outcomes. To fully validate any recommended injury threshold, it is essential to not only assess whether the predicted injury locations align with clinical findings but also consider cases where inappropriate thresholds may lead to overestimation or underestimation of injury areas. This issue becomes evident only when prediction for the entire brain is performed.

In light of this, the present study focuses on the MPS injury criterion and aims to develop a novel data-driven method, grounded in the stationary value principle, to determine an objective injury threshold. The rationality of this threshold is assessed by comparing entire brain injury predictions with CT images from a real clinical case. Additionally, an occipital impact scenario is designed to apply the new threshold in predicting entire brain injury and to analyze the correlation between the predicted injury areas and observed memory impairment.

## 2. Methods

**Figure 1:**
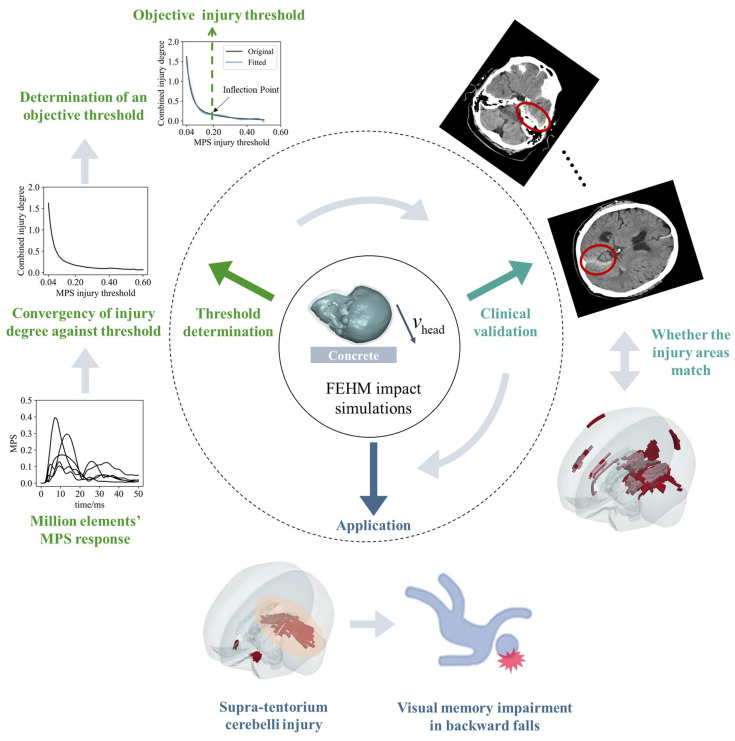
An objective injury threshold for the MPS criterion is determined by FEHM impact simulations, with clinical validation and its application. (1) Threshold determination: the converged relationship between the combined injury degree and the injury threshold is derived from the MPS–time curves of millions of brain tissue elements in finite element simulations (graphically illustrated by the MPS–time curves of five brain finite elements). The stable inflection point in the curve indicates an objective threshold. (2) Clinical validation: the threshold is validated by comparing the injury areas predicted by the FEHM with CT images from a clinical case. (3) Application: the threshold is applied to backward falls, with the predicted supra-tentorium cerebelli injury offering insights into the biomechanical mechanism of visual memory impairment.

In this study, an FEHM was used to simulate head impacts with the aim of determining an objective injury threshold for the MPS criterion in brain tissue. The method consisted of three main parts: (1) threshold determination, (2) clinical validation, and (3) application to backward falls. As illustrated in Figure 1, a large dataset was first established through multiple non-injurious head impact simulations under various conditions. A combined relationship between injury degree and injury threshold was built by analyzing the responses of millions of brain tissue elements. Based on the stationary value principle, a hypothesis was proposed, and the objective injury threshold was determined from the converged relationships. This data-driven method significantly differs from the previously mentioned clinical case-driven approach. The threshold was validated by comparing the predicted injury areas with CT images from a TBI case. Finally, the validated threshold was applied to backward falls, predicting whole-brain injury through finite element simulations of the designed occipital impacts. Combined with the relevant neuroscience literature, the biomechanical mechanism underlying visual memory impairment due to occipital impact is explored.

### 2.1. Establishment of a Large Simulation Dataset

**Figure 2:**
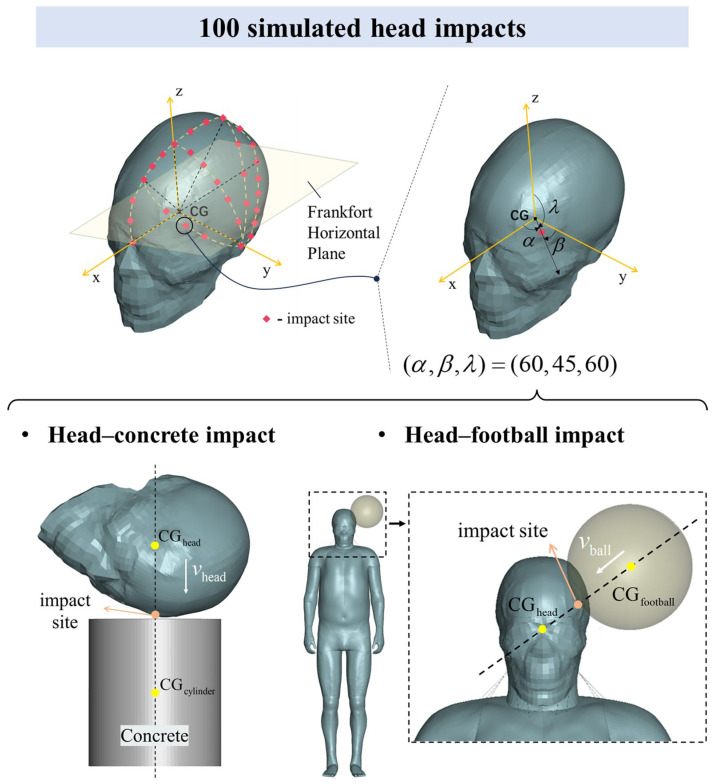
A total of 100 head impacts from MPS response data were used to construct a large dataset. There were 50 head–concrete impacts and 50 head–football impacts. Each impact case was uniquely determined by the impact site, impact velocity, and impact type. The simulation included 30 impact sites (red dots), covering the left side of the head. A right-handed Cartesian coordinate system was established with the center of gravity of the head (${\mathrm{CG}}_{\mathrm{head}}$) as the origin and the XY plane parallel to the Frankfort horizontal plane. Based on this coordinate system, the impact site was represented by the direction angle $\left(\alpha ,\;\beta ,\;\lambda \right)$ of its position vector. The impact site represented by (60, 45, 60) was taken as an example. Then, based on the line connecting the impact site and ${\mathrm{CG}}_{\mathrm{head}}$, the centers of gravity of the concrete cylinder block and the football ${(\mathrm{CG}}_{\mathrm{cylinder}}{,\;\mathrm{CG}}_{\mathrm{football}})$ were determined so that the three points were collinear (dashed line). The impact velocity is shown by the white arrow. The impact velocities of the head hitting concrete and the football hitting the head are $v^{\mathrm{head}}$ and $v^{\mathrm{ball}}$, respectively.

A large simulation dataset was constructed using the Total Human Model for Safety (THUMS), a finite element human body model developed in collaboration between Toyota Motor Corporation, Toyota, Japan and Toyota Central R&D Labs., Inc., Nagakute, Japan [34,35]. As shown in Figure 2, we designed 100 head impact simulations, covering the left side of the head. These included 50 head–concrete impacts [36,37] and 50 head–football impacts [38,39], using the anatomical Frankfort horizontal plane as a reference. This plane is defined by the line connecting the lowest point of the orbit’s lower edge to the upper edge of the ear canal. In the first scenario, the head model strikes a stationary concrete block at an initial velocity $v^{\mathrm{head}}$, representing a hard impact. In the second, a football, moving at an initial velocity $v^{\mathrm{ball}}$, impacts the head of the whole-body model, representing a soft impact. Excluding penetrating impacts, these two types cover most typical head impact scenarios.

The head model is derived from the THUMS AM50 V6 validation set, while the whole-body model is derived from the THUMS pedestrian V4.02. The head–football impact settings reference our previous work [39]. For each simulated impact case in LS-DYNA R10.1.0 (Livermore Software Technology Co., Livermore, CA, USA), the calculation time was set to 50 ms, sufficiently long to capture the peak MPS of each brain element during the impact. Subsequently, we aggregated the peak MPS values of all brain elements across all impact cases to create a large dataset.

Prior to the simulation, we made modifications to the geometry of the frontotemporal region to more accurately reflect the actual structure of the human brain. For a comprehensive understanding, details of the structural modifications, materials used in the simulations, and interaction parameters are provided in Appendix A. Based on the consistency of intracranial responses—such as intracranial pressure and relative brain displacement—between THUMS and cadaver experiments, the dynamic responses simulated by the model exhibit a certain degree of objective validity [35].

Each impact case was uniquely determined by the impact type, impact site, and impact velocity. The impact site descriptions were based on a right-handed Cartesian coordinate system, with the head’s center of gravity (${\mathrm{CG}}_{\mathrm{head}}$) serving as the origin, and the XY plane aligned parallel to the Frankfort horizontal plane. Up to 30 impact sites on the left side of the head were selected, with each site’s location precisely mapped using direction angles $\left(\alpha ,\;\beta ,\;\lambda \right)$ from the position vector. The centers of both the concrete cylinder block and the football were aligned with a dashed line that passes through the impact site and the head’s center of gravity. For most impact sites, only one velocity direction was applied, where the velocity vector is parallel to this dashed line, referred to as a normal impact. In some typical impact positions, such as the frontal impact site (45, 90, 45), both normal and oblique impacts were designed. For the impact velocity, our goal was for most impact cases to fall within the range of non-injury to severe brain injury at a given velocity. Based on previous accident reconstructions of real head hard-impact cases [2,40] and research on football-related head impacts [38,39], we set the impact velocities for the head–concrete and head–football impacts to 3 m/s and 35 m/s, respectively. Due to limitations in computational time and storage capacity, the impact velocity was not considered as a variable for deeper analysis in this study. A detailed description of the impact cases can be found in Appendix B.

**Figure A2:**
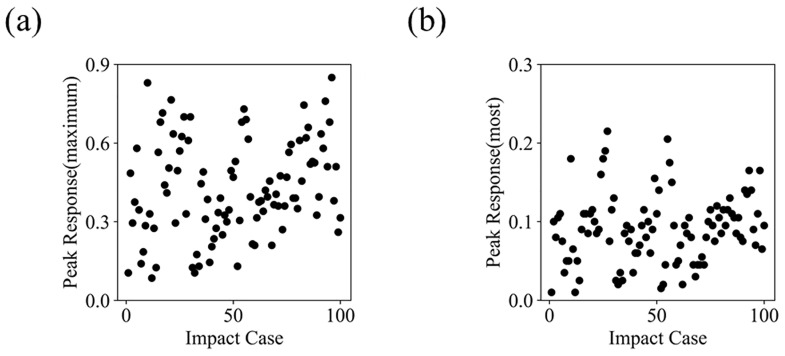
Distribution characteristics of peak MPS in the large simulation dataset. (**a**) The distribution of the maximum peak values across all cases, with a range of 0.08 to 0.85, and an average value (standard deviation) of 0.43 (0.18). (**b**) The distribution of the most frequently occurring peak values, ranging from 0.01 to 0.21, with an average value (standard deviation) of 0.09 (0.04).

To ensure the comprehensiveness of the dataset and avoid an overly concentrated distribution of peak MPS values, we analyzed the distribution characteristics (see Figure A2 in Appendix B). Additionally, before calculating the injury threshold, all impact cases were evaluated to confirm that potential brain injuries were limited to closed head injuries. By applying the established internal energy threshold of 2.2 J, which corresponds to a 50% risk of skull fracture [41], we verified that all cases remained within the expected range.

### 2.2. Determination of an Objective Injury Threshold for MPS Criterion

In the “clinical case-driven” method, the most reasonable injury threshold is determined by the binary relationship between the maximum peak MPS in the region of interest and the occurrence of injury. The principle of this method assumes that an objective MPS injury threshold exists. This allows for determination of whether any brain tissue deformation at any location results in injury, and the degree of injury can be quantified accordingly.

Here, we define $D_{i}^{\mathrm{ele}}$ as the injury degree of an element i, representing functional impairment. It is calculated based on the element’s peak MPS ($P_{i}$) and the injury threshold ($T_{\mathrm{inj}}$) according to Equation (1),

(1) $$D_{i}^{\mathrm{ele}}=\left\{\begin{array}{c} \frac{P_{i}-T_{\mathrm{inj}}}{T_{\mathrm{inj}}},P_{i}>T_{\mathrm{inj}}\\ 0,P_{i}\leq T_{\mathrm{inj}} \end{array}\right.$$

when $P_{i}\leq T_{\mathrm{inj}},\;D_{i}^{\mathrm{ele}}=0$, and we refer to the element as an “uninjured element”. Conversely, if $P_{i}>T_{\mathrm{inj}},\;D_{i}^{\mathrm{ele}}>0$, the element is classified as an “injured element”.

**Figure 3:**
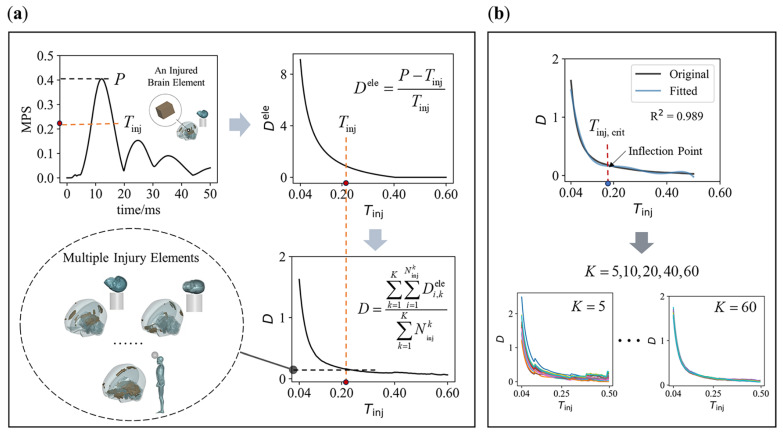
The relationship of combined injury degree D against injury threshold $T_{\mathrm{inj}}$ and its $T_{\mathrm{inj},\mathrm{crit}}$. (**a**) Extract the peak MPS P from the element’s time histories, and calculate the element injury degree based on P and the given injury threshold $T_{\mathrm{inj}}$ (highlighted red dot). Then, a combined injury degree D of all injured elements under K impact cases is calculated. As $T_{\mathrm{inj}}$ increases, $D^{\mathrm{ele}}$ and $N_{\mathrm{inj}}^{k}$ decrease, forming a relationship between D and $T_{\mathrm{inj}}$; (**b**) 5th-order polynomial fitting is used to solve for the $T_{\mathrm{inj},\mathrm{crit}}$ of a $D-T_{\mathrm{inj}}$ curve (*K* = 5, 10, 20, 40, 60).

By treating $T_{\mathrm{inj}}$ as the independent variable, we can observe the variation in $D_{i}^{\mathrm{ele}}$ with respect to $T_{\mathrm{inj}}$, as shown in Figure 3a. As $T_{\mathrm{inj}}$ increases, $D_{i}^{\mathrm{ele}}$ decreases until the element is classified as “uninjured”. Here, we propose a hypothesis: near the objective injury threshold, the absolute rate of change in the injury degree is minimized. This idea is based on the stationary value principle, which suggests that, under certain conditions, the derivative of a function at an extreme point (maximum or minimum) is zero [42]. In physics, this principle is often used to identify a system’s stable state. In our hypothesis, the injury degree corresponding to the objective injury threshold represents a stable state, reflected by the minimum absolute rate of change.

The relationship between the injury degree and the injury threshold of a single element does not fully support this hypothesis. Therefore, we need to consider the overall effect across multiple elements under K impact cases. To achieve this, we define a combined injury degree D as follows:

(2) $$D=\frac{\sum_{k=1}^{K}\sum_{i=1}^{N_{\mathrm{inj}}^{k}}D_{i,k}^{\mathrm{ele}}}{\sum_{k=1}^{K}N_{\mathrm{inj}}^{k}}$$

where $D_{i,k}^{\mathrm{ele}}$ represents the injury degree of element i in impact case k, and $N_{\mathrm{inj}}^{k}$ is the number of injured elements in case k based on the threshold $T_{\mathrm{inj}}$. For any given number K of impact cases, $\sum_{k=1}^{K}N_{\mathrm{inj}}^{k}$ is the total number of injured elements across all K cases, and D is the average injury degree of all injured elements. As the threshold $T_{\mathrm{inj}}$ increases, D changes correspondingly. This results in what we call the “combined relationship” between the combined injury degree and the injury threshold, as shown in Figure 3a.

Our goal is to identify the threshold $T_{\mathrm{inj}}$ that minimizes the absolute rate of change in D in the combined relationship, or in other words, to select the $T_{\mathrm{inj}}$ corresponding to the curve’s inflection point (hereinafter referred to as $T_{\mathrm{inj},\mathrm{crit}}$, as illustrated in Figure 3b). An important question arises: how large must K be for the resulting $T_{\mathrm{inj},\mathrm{crit}}$ to reliably represent the objective injury threshold? In this context, $T_{\mathrm{inj},\mathrm{crit}}$ should converge to a consistent value, regardless of the specific cases included. To address this, we analyzed how $T_{\mathrm{inj},\mathrm{crit}}$ varies with different values of K (set to 5, 10, 20, 40, and 60), as illustrated in Figure 3b. Specifically, we randomly selected K cases from a total of 100 impact cases to construct the combined relationship, and for each K, we generated 20 combined relationships.

By fitting the combined relationship using a polynomial, we can determine $T_{\mathrm{inj},\mathrm{crit}}$ for each fitted curve (as shown in Figure 3b). This process involves three key factors: (1) the order of the polynomial; (2) the goodness of fit $R^{2}$; and (3) the fitting interval. To avoid overfitting and excessive amplification of high-order fluctuations in the original curve, we used a 5th-order polynomial for the fitting process [43,44], with $R^{2}\geq 0.98$ as the criterion for a successful fit. Additionally, to determine the most appropriate fitting interval, we fit the two combined relationships—derived from 50 head–concrete impacts and 50 head–football impacts, respectively—using multiple fitting intervals. We believe that a reasonable fitting interval should minimize the error of $T_{\mathrm{inj},\mathrm{crit}}$ in two curves while maintaining the largest possible fitting interval. If a 2% error is deemed acceptable, the largest fitting interval that meets this criterion is 0.04–0.5.

### 2.3. Clinical Case and Corresponding Occipital Impact

This section aims to verify the rationality of the objective injury threshold by referencing a specific clinical case. The inclusion criteria for selecting clinical case reports from the literature are as follows: (1) closed head injuries without explosive trauma or localized skull deformation; (2) a clearly described impact scenario that allows for reasonable estimation of the impact position, direction, and speed; and (3) comprehensive injury descriptions, including relatively complete CT or MRI imaging. Based on these criteria, we selected a clinical case involving an occipital impact after a fall [45]. In this case, a 46-year-old male fell from a short distance, landing on his back, with his head striking the ground. Brain CT scans revealed hemorrhages and contusions in the left supratentorial cerebellum, falx cerebri, and temporal lobe. This was a body-buffered backward fall, meaning that most of the kinetic energy was absorbed by the hips and hands as they made initial contact with the ground, with the head striking the ground afterward [46,47].

Since the case did not provide specific details about the head impact, it is not possible to reconstruct the exact impact scenario, unlike reconstructions based on sports event footage [33] or traffic accident data [48]. However, if MPS is a reliable injury variable, impact cases within a reasonable range of impact site and velocity (both magnitude and direction) should exhibit injury distribution patterns similar to the actual injury. In terms of impact velocity, an experimental study on backward falls in the elderly, where body cushioning was present, recorded a vertical head impact velocity of 1.67 (±0.42) m/s from a height of 5 cm [49]. In contrast, simulations using MADYMO (Mathematical Dynamic Models) for falls without body cushioning reported head impact velocities exceeding 3 m/s [11]. In this study, we estimated the head impact velocity based on two real-life backward falls (see Appendix C for details), and the vertical and horizontal velocities are listed in Table 1. From these estimates, we infer that the vertical impact velocity of the head with body cushioning may range from 2 to 3 m/s, while the horizontal velocity may range from 0 to 2 m/s.

**Figure 4:**
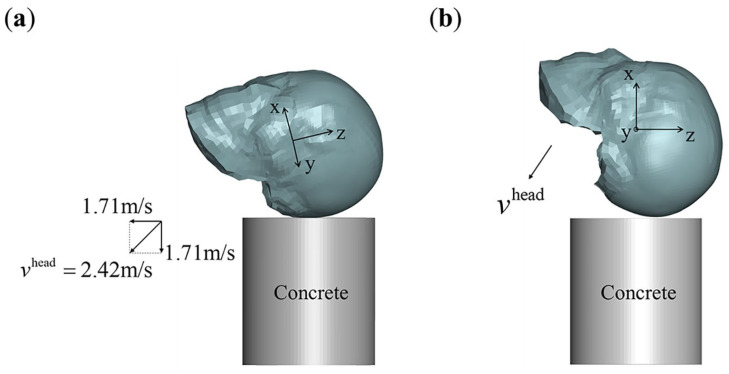
Occipital impacts for clinical case-based validation and application. (**a**) Left occipital impact that may correspond to the clinical case, with an impact velocity $v^{\mathrm{head}}=2.42\;m/s$. The head posture is uniquely determined by the coordinate system shown in the figure. In this posture, the total velocity $v^{\mathrm{head}}$ can be decomposed into 1:1 vertical and horizontal component velocities; (**b**) designed occipital impacts for application. In this case, the velocity component in the y-direction is set to zero, and the total velocity is composed of components in the x- and z-directions.

Figure 4a presents a possible left occipital impact scenario corresponding to the clinical case, where the total head velocity $v^{\mathrm{head}}=2.42\;m/s$ (representing the head’s landing velocity from a 0.3 m free fall) is shown. This velocity can be decomposed into equal vertical and horizontal components, as illustrated in Figure 4a. The velocity components in the three vertical directions are listed in Table 1.

Next, we used the objective injury threshold to predict the entire brain injury area for the left occipital impact and compare the predicted injury area with different sections from the CT images. The referenced CT image sections are parallel to the skull base [50]. Accordingly, we cut the corresponding FEHM sections using planes parallel to the skull base and determined the specific FEHM sections by comparing the distribution characteristics of the brain tissue, ventricles, and skull in both the FEHM and CT sections.

### 2.4. Occipital Impacts for Application

Clinical reports of occipital impacts from backward falls have documented brain dysfunctions, including memory, cognitive, and auditory impairments [51,52]. Similarly, memory impairment has been observed in some occipital impacts resulting from rear-end collisions [53,54]. Given the frequent occurrence of memory impairment in clinical reports of occipital impacts, this study focuses on assessing the correlation between memory impairment and injury predictions across the entire brain. Based on previous estimates of occipital impact velocities in backward falls with body cushioning, we designed seven impact velocity combinations, as outlined in Table 1. A schematic diagram of these impacts is shown in Figure 4b.

**Figure 5:**
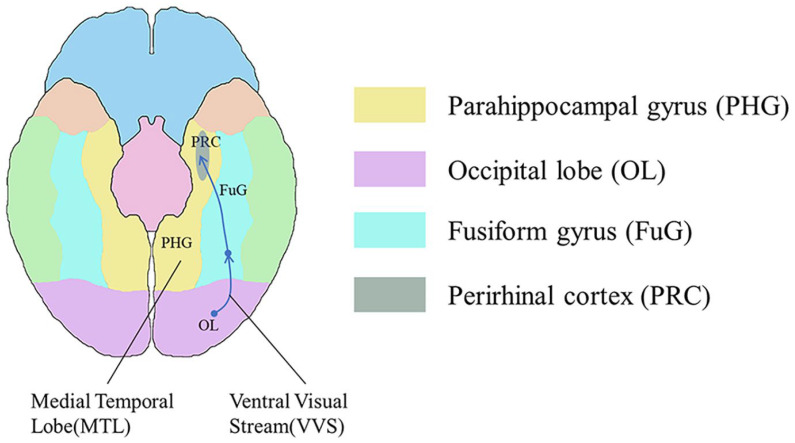
The representational–hierarchical framework for visual perception and memory functions is constituted by the ventral visual stream (VVS) and the medial temporal lobe (MTL). In the ventral view with the cerebellum and brainstem removed, the corresponding regions of the parahippocampal gyrus (PHG) in yellow, the occipital lobe (OL) in purple, the fusiform gyrus (FuG) in blue, and the perirhinal cortex (PRC) in gray are highlighted. In addition, the green, orange, and blue colors in the figure represent the corresponding areas of the inferior temporal gyrus, temporal pole, and frontal lobe, respectively.

Figure 5 illustrates a representational–hierarchical framework comprising the ventral visual stream (VVS) and the medial temporal lobe (MTL), both recognized as key areas for visual perception and memory functions [55]. In this study, we reference this framework in relation to memory function. The MTL primarily includes the hippocampus, along with the closely associated entorhinal, perirhinal, and parahippocampal cortices [56]. The VVS begins in the primary visual cortex and extends along the ventral side of the temporal lobe towards the piriform cortex.

## 3. Results

### 3.1. The Objective Injury Threshold

**Figure 6:**
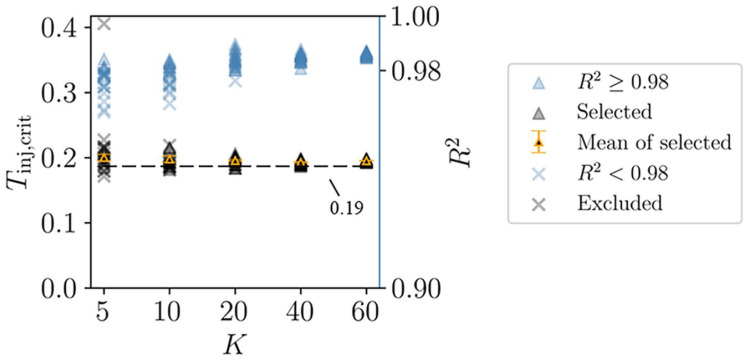
An objective injury threshold is determined through the convergence of combined relationships. The selected $T_{\mathrm{inj},\mathrm{crit}}$ and the excluded $T_{\mathrm{inj},\mathrm{crit}}$ are divided from the $T_{\mathrm{inj},\mathrm{crit}}$ of all fitting curves with $R^{2}=0.98$ as the boundary. It can be seen that if K≥20, the mean of the selected $T_{\mathrm{inj},\mathrm{crit}}$ fluctuates between 0.19 and 0.2. The error bar is the standard error.

Figure 6 shows the $T_{\mathrm{inj},\;\mathrm{crit}}$ and $R^{2}$ values of the fitting curves for all combined relationships. Some $R^{2}$ values fall below 0.98, meaning the corresponding $T_{\mathrm{inj},\;\mathrm{crit}}$ values should be excluded. As K increases, $T_{\mathrm{inj},\;\mathrm{crit}}$ gradually stabilizes, and the corresponding $R^{2}$ approaches 1. Notably, when K≥20, the mean of the selected $T_{\mathrm{inj},\;\mathrm{crit}}$ values fluctuates between 0.19 and 0.2, reflecting the convergence characteristics of the combined relationships. Consequently, we adopted 0.2 as the objective injury threshold for MPS.

### 3.2. Injured Areas in FEHM and CT Images

**Figure 7:**
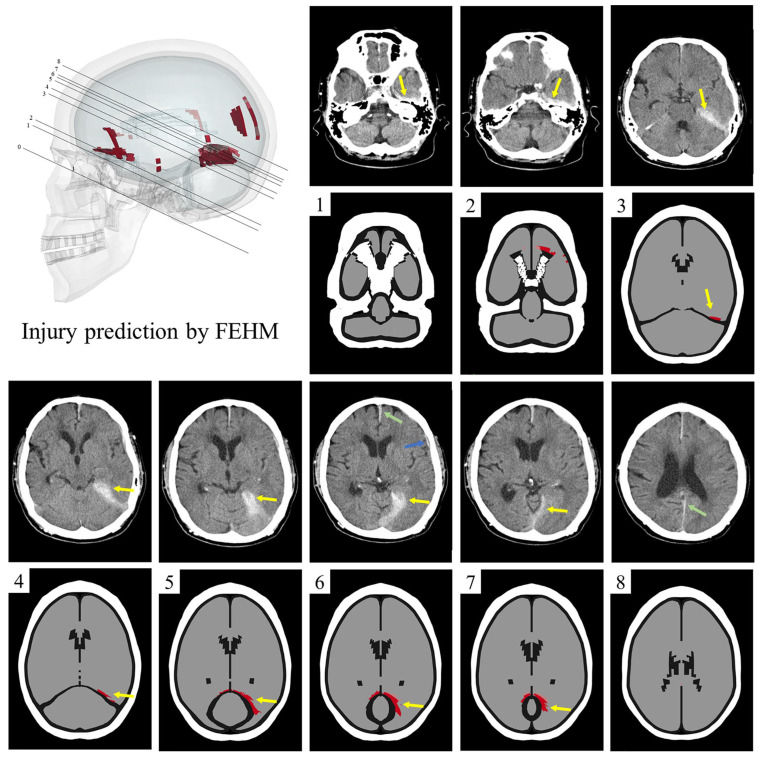
The comparison of injury areas in FEHM and CT images. Clinically, there is hematoma or contusion from the temporal lobe to the upper tentorium (yellow arrow), as well as hemorrhage in the falx cerebri, between the cerebral hemispheres (green arrow), and contusion in the sylvian fissure (blue arrow). The FEHM injury predictions of the corresponding sections are below the CT images. In sections 3–7, the yellow arrow indicates the injury above the tentorium, which corresponds to the clinical results. In addition, injuries in the orbitofrontal region are also indicated (section 2), which are not present in CT images. Hemorrhaging between the cerebral hemispheres and contusions in the sylvian fissure are visible only in the CT images (sections 6 and 8).

The CT image sections of the clinical case are parallel to the skull base. The FEHM section corresponding to the skull base is labeled as section 0 in Figure 7. In the other eight FEHM sections, the skull is marked in white, cerebrospinal fluid and air in black, and uninjured brain tissue in gray. The injured areas, which appear as high-density shadows in the CT images, are marked in red (gray matter) and pink (white matter) in the FEHM.

In sections 3–7, both the CT images and FEHM predictions show extensive injury above the tentorium. In sections 1 and 2, the CT images indicate a high-density shadow in front of the petrous part of the temporal bone, which is not predicted by the FEHM. Additionally, in sections 2, 6, and 8, there are discrepancies between the injured areas in the CT images and those predicted by the FEHM. Hemorrhaging between the cerebral hemispheres and contusions in the sylvian fissure are visible only in the CT images (sections 6 and 8), while orbitofrontal injury is predicted solely by the FEHM (section 2). The FEHM also predicts minor cortical and corpus callosum injuries.

### 3.3. Injured Predictions in Occipital Impacts

**Figure 8:**
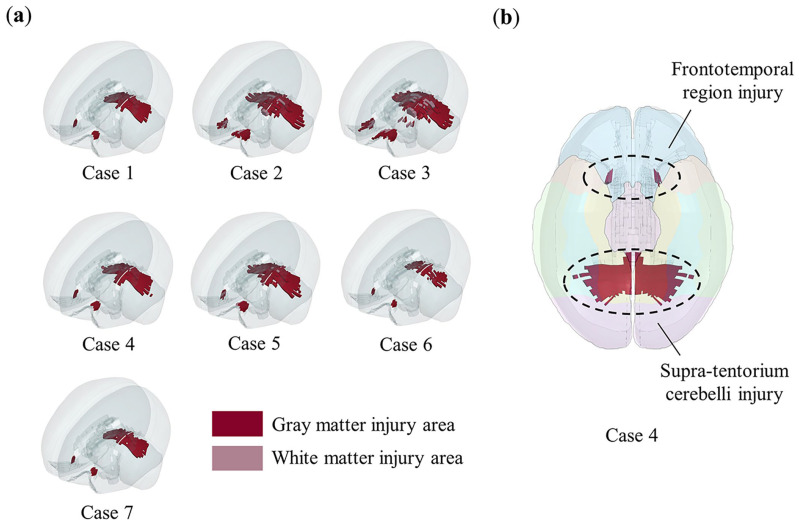
The injury predictions in designed occipital impacts and its correlation with memory impairment. Red color indicates gray matter element injury, pink color indicates white matter element injury, and elements not highlighted are not injured. (**a**) The injury areas predicted by the objective injury threshold (0.2) in all occipital impacts. The injury prediction distribution of all occipital impacts is similar, mainly concentrated in two areas: frontotemporal region and supra-tentorium cerebelli. Notably, in case 2 and case 3, where vertical velocities reached up to 3 m/s, minor subcortical white matter injuries are observed; (**b**) in case 4, as shown by comparing with Figure 5, both injury areas (frontotemporal region and supra-tentorium cerebelli) fall within the representational–hierarchical framework composed of the MTL and VVS.

Using the objective injury threshold, we predicted the injured areas of the brain for the occipital impacts designed in Table 1. The results are shown in Figure 8a, where the injury predictions for all occipital impacts focus on two main regions: the frontotemporal region and the supra-tentorium cerebelli. For example, in case 4, Figure 8b shows that the predicted injury areas align with the regions involved in the representational hierarchy framework.

## 4. Discussion

Tissue-level injury variables and their thresholds are crucial in linking kinematic data to microscopic injuries. This study determined an objective MPS injury threshold for brain injury using a novel data-driven method. Compared to other thresholds, the one recommended here is more robust in terms of both its physical significance and validation. In applying this new threshold to predict injuries from occipital impacts, special attention must be paid to supra-tentorium cerebelli injury, particularly in cases involving visual memory impairment.

### 4.1. Objectivity Analysis for the New Threshold

In general, for brain tissue subjected to any head impact, as the injury threshold $T_{\mathrm{inj}}$ increases, the injury degree $D^{\mathrm{ele}}$ of each element decreases, as shown in Figure 3a. When considering the relationship between the combined injury degree D of a large number of elements and the injury threshold $T_{\mathrm{inj}}$, D also tends to decrease as $T_{\mathrm{inj}}$ increases, as illustrated in Figure 3a. As shown in Figure 3b, with an increasing number K of impact cases combined, the combined curve converges. This convergence indicates that as K increases, the inflection point of the curve stabilizes, and the threshold corresponding to the inflection point $T_{\mathrm{inj},\mathrm{crit}}$ fluctuates around a specific value.

Figure 6 shows that when K=20, the $T_{\mathrm{inj},\mathrm{crit}}$ values from all combined relationships converge closely (the average value is 0.195, with a standard error of 0.001). Since these combined relationships are formed by randomly selecting 20 impact cases from a total of 100, the independence of these relationships is ensured. This result illustrates that when a sufficient number of impact cases are considered (K≥20), the curve describing the relationship between the combined injury degree D and the injury threshold $T_{\mathrm{inj}}$ becomes stable, and the inflection point of the curve barely changes. This indicates that the threshold is determined objectively. The threshold obtained through this method no longer depends on a specific impact case but requires a sufficient number of cases. This is why we refer to this method as data-driven.

An objective brain injury threshold can be derived from the data-driven method due to several key factors. First, the assumption that the absolute rate of change in injury degree is minimized near the objective injury threshold is inherently reasonable. Additionally, the dataset used in this study, as well as the definition of injury degree, meets the necessary requirements. The dataset must be comprehensive, meaning the peak responses of all elements should cover a wide range and be sufficiently dispersed. As shown in Figure A2, the mean and standard deviation of the maximum peak values for each impact case in the dataset are 0.43 and 0.18, respectively, while the mean and standard deviation of the most concentrated peak responses are 0.09 and 0.04, respectively. These characteristics reflect the comprehensiveness of the dataset.

Furthermore, the definition of element injury degree, based on peak response, is also reasonable. All head impacts in this study were simulated under non-injury conditions, meaning the brain tissue material model did not account for injury. After obtaining the peak response of an element, the injury degree is determined by the ratio of the difference between the peak response and the threshold to the threshold itself. This definition is valid when the strength of the brain tissue material has not been significantly reduced, ensuring that the error between the calculated and actual injury degrees is negligible. However, it is worth noting that a small number of larger MPS peak responses in the dataset (up to 0.85) do exist. Future research will aim to further analyze this, potentially limiting the scope of applications for this method. Investigating whether an objective injury threshold can be derived using the same data-driven method for different brain tissue mechanical parameters is an interesting avenue for future exploration.

### 4.2. Rationality of the New MPS Injury Threshold

Table 2 lists the MPS injury thresholds for different brain regions obtained using the “clinical case-driven” method in previous studies. When the maximum peak response in a given region exceeds these thresholds, there is a 50% probability of concussion. Concussion, a form of uncomplicated mTBI, is clinically characterized by normal routine imaging but impaired brain function [57]. As shown in Table 2, there are significant variations in the thresholds for different regions of interest across various clinical datasets and computational models. Despite these differences, the thresholds imply the strain tolerance level of brain tissue related to concussion, generally ranging from 0.13 to 0.27. However, no single threshold has been definitively proven to be more valid than the others in previous studies. The significance of our work lies in determining a specific threshold within this range and validating it through entire brain injury prediction.

Figure 7 shows the predicted injury areas of the entire brain under the left occipital impact, including the supra-tentorium cerebelli, left orbitofrontal region, and small areas of the cortex and corpus callosum. The accurate prediction of supra-tentorium cerebelli injury in multiple sections by the FEHM demonstrates its capability to predict real injuries when combined with the new injury threshold we established. Since the degree of injury in other areas is lower than that in the supra-tentorium cerebelli, we speculate that these regions may have only sustained functional impairment, which was not detected in the CT images. It is worth noting that frontotemporal injuries from occipital impacts are often classified as contrecoup injuries, typically caused by intracranial pressures and relative skull/brain movements [60]. However, our injury prediction suggests that the frontotemporal injury in the left occipital impact results primarily from large shear deformations. Regarding the contusion observed in the anterior petrous part of the temporal bone in the CT image but not predicted by the FEHM, we hypothesize that this could be due to anatomical differences—specifically, the patient’s skull base may be more prominent than that of the FEHM, making this region more prone to injury.

**Figure 9:**
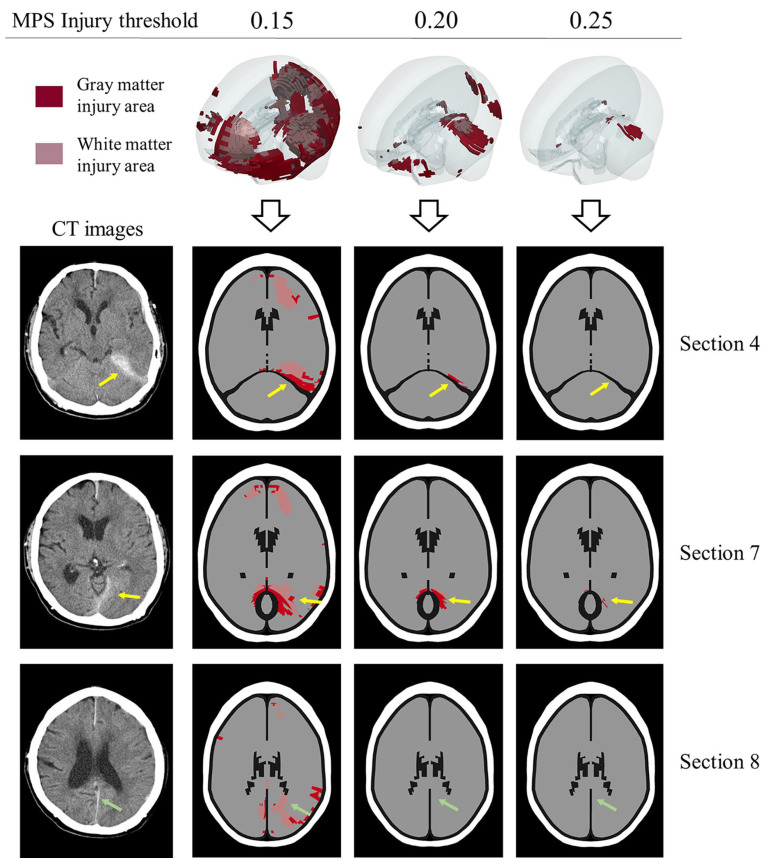
The rationality of the MPS objective injury threshold (0.2) in predicting entire brain injury is evaluated. The prediction effects of thresholds 0.15, 0.2, and 0.25 are compared with CT images. Red color indicates gray matter element injury, pink color indicates white matter element injury, and elements not highlighted are not injured. The yellow arrow and green arrow indicate the injuries in the supra-tentorium cerebelli and between the cerebral hemispheres, respectively. The prediction area of 0.15 is significantly larger, and the prediction area of 0.25 is significantly smaller. In comparison, the prediction effect of 0.2 is more consistent with the CT images.

In previous work, the main approach for verifying FEHM injury prediction based on clinical cases has focused on whether the FEHM can accurately predict clinically detected injuries. Fahlstedt et al. quantitatively analyzed the consistency between injury areas predicted by medical images and FEHM by reconstructing three independent accidents [61]. Similarly, Yuan et al. reconstructed a skiing accident and found that the location and distribution predicted by the FE model closely matched the clinical diagnosis in the cross-sections where hematoma and micro-bleeding occurred [33]. However, these studies lacked further analysis of the predicted entire brain injury area, leaving room for potential overestimation or underestimation of injury areas due to an unreasonable threshold. As shown in Figure 9, we compared the predicted injury areas using three different thresholds (0.15, 0.2, and 0.25) with CT images. A threshold of 0.15 predicted an excessively large injury area compared to the clinical diagnosis, while a threshold of 0.25 was too high, failing to accurately predict real injuries in three sections. In contrast, the injury areas predicted by a threshold of 0.2 were more consistent with the clinical diagnosis. In summary, this study is the first to conduct a comprehensive and in-depth analysis of the rationality of predicted injury areas across the entire brain, which is significant for using finite element analysis to assist in the clinical diagnosis of brain injuries, especially for patients with closed head injuries but no visible brain contusions. Additionally, in the context of designing protective equipment and assessing risk in sports or automobile accidents, our recommended threshold can help more accurately determine product safety factors. However, further testing is needed to assess the ability of the recommended threshold to distinguish between clinical cases with and without clinically observed brain injuries. Evaluating the sensitivity, specificity, and overall accuracy of the threshold in clinical validation will be a key focus in future collaborations with clinicians.

In addition to comparing the threshold with clinical cases, it is important to also consider the tissue’s functional tolerance, as observed in in vitro tissue experiments. Kang et al. studied the variation in electrophysiological function in relation to tissue-level injury parameters (strain and strain rate) for rat hippocampal tissue, indicating that a strain of 0.2 at a strain rate of 10 s^−1^ caused the duration of neural events to peak [62]. This suggests that, at this strain rate, a strain of 0.2 optimally activates neuronal ion channels and signal transduction mechanisms, while strains beyond 0.2 may lead to neuronal dysfunction, reducing the duration of neural events. Bain et al. conducted uniaxial stretching on guinea pig optic nerves to induce axonal injury and performed logistic regression on the relationship between morphological injury and strain, determining an optimal strain threshold of 0.21 for white matter morphological injury [63]. While these tissue strain tolerances suggest that 0.2 may be a reasonable threshold for functional impairment, it is important to note that these in vitro experiments involve a simple stress state. In contrast, real head impacts involve much more complex stress states. In the future, it will be crucial to employ advanced 3D technology [64] to better model the real head structure, while incorporating impact experiments [65] or simulations with electrophysiological measurements.

### 4.3. Visual Memory Impairment and Supra-Tentorium Cerebelli Injury

For patients who have experienced an occipital impact, clinical observations often report memory impairments [51,52,53,54], including visual memory impairment [53,66], even though the relevant injury areas are not typically confirmed through conventional CT or MRI examinations. Our analysis in Figure 8 indicates that occipital impacts can result in injuries to the frontotemporal region and the supra-tentorium cerebelli, both of which are associated with memory function. However, as we emphasize, these are functional injuries identified through the functional injury threshold, making them difficult to detect compared to structural injuries like hematomas or contusions.

The representational–hierarchical framework demonstrates that in regions composed of the ventral visual stream (VVS) and medial temporal lobe (MTL), different levels of representational complexity allow the corresponding brain regions to independently process memory and visual information perception. Therefore, we infer that the supra-tentorium cerebelli injury observed in occipital impacts (Figure 8b) is linked to visual memory impairment, a condition less frequently diagnosed in clinical practice. Supra-tentorium cerebelli injuries are rare in clinical cases [67,68] and may be difficult to diagnose, particularly in unfamiliar situations [69]. We suggest that clinicians should pay special attention to the supra-tentorium cerebelli during examinations when occipital impacts are accompanied by visual memory impairment and administer targeted treatments as necessary.

## 5. Conclusions

In conclusion, we determined that 0.2 is an objective MPS injury threshold through a novel data-driven method. The predicted injury area based on this threshold closely matches the major injury area (the supra-tentorium cerebelli) observed in the clinical case, demonstrating both the validity of the threshold and the feasibility of the data-driven method. We emphasize that occipital impacts can lead to supra-tentorium cerebelli injuries. Considering that this type of injury is relatively rare in clinical practice, yet visual memory impairment associated with it is more frequently reported, we recommend that clinicians give careful attention to potential supra-tentorium cerebelli injuries when visual memory impairment follows occipital impacts, to guide prognosis and appropriate treatment.

## Figures and Tables

**Table 1:** Summarization of occipital impact velocities.

	Case	$\left|v_{x}^{head}\right|(m/s)$	$\left|v_{y}^{head}\right|(m/s)$	$\left|v_{z}^{head}\right|(m/s)$
Video analysis	1	3.17	0	1.72
	2	2.37	0	0.75
Left occipital impact	-	0.982	0.775	2.076
Designed occipital impacts	1	3	0	0
	2	3	0	1.5
	3	3	0	2
	4	2.5	0	1.5
	5	2.5	0	2
	6	2	0	1.5
	7	2	0	2

**Table 2:** Summarization of MPS injury thresholds.

Injury	Region	MPS Threshold	Reference
50% Concussion	Corpus callosum	0.21	[25]
	Gray matter	0.26	
	Corpus callosum	0.2	[16]
	Corpus callosum	0.15	[58]
	Thalamus	0.13	
	White matter	0.26	
	The entire brain	0.24	[26]
	The entire brain	0.27	[59]
Functional impairment	The entire brain	0.2	This study

**Figure A1:**
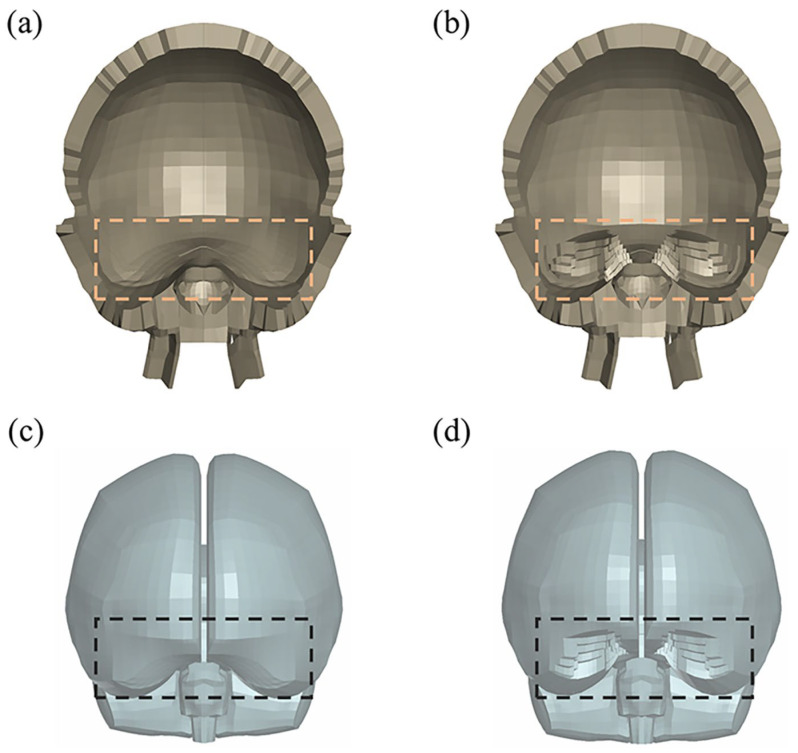
Modifications to the geometric structure of the THUMS head model in the orbital frontal and temporal pole regions (dashed boxes). (**a**) Skull before modification; (**b**) skull after modification; (**c**) brain tissue before modification; (**d**) brain tissue after modification.

**Figure A3:**
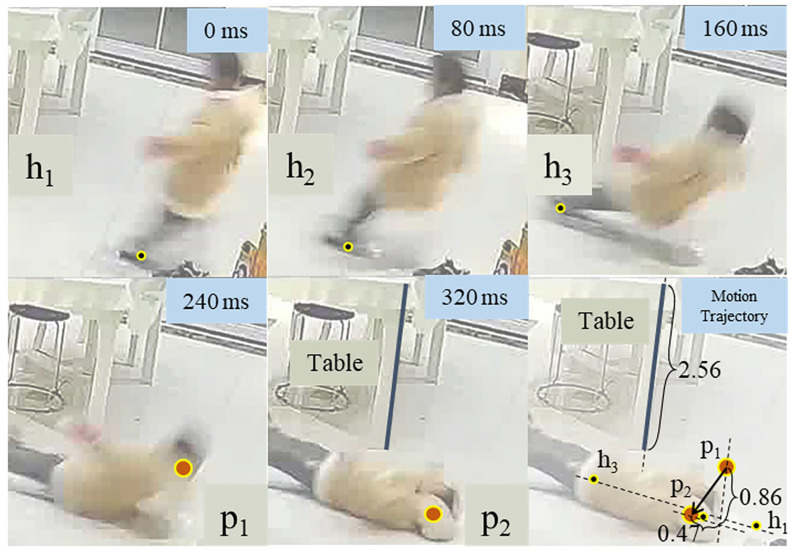
Using frames from the incident video, the trajectory of the head moving downwards and horizontally is tracked by $p_{1}$, $p_{2}$ (dots filled with red) and $h_{1}$, $h_{2}$, $h_{3}$ (dots filled with black), respectively. Assuming the actual length of the table is 75 cm (2.56 in the picture), the actual displacement in two directions (0.86 vertically and 0.47 horizontally) could be calculated.

## Data Availability

The data and primary code used in the current study are publicly available at the following repository: https://github.com/Zhang-Yu-Ting/Data-Driven-Brain-Injury-Threshold, accessed on 10 September 2024.

## Appendix A

Figure A1 illustrates the modifications made to the geometric structure of the THUMS head model. The material properties of the head model, concrete block, and football are detailed in Table A1. The skin of the head model, as well as the inner and outer surfaces of the skull, are set with AUTOMATIC_SINGLE_SURFACE contact. The cerebrospinal fluid, meninges, and brain tissue elements are modeled with shared nodes, which is equivalent to tied contact. The contact between the head skin and the concrete block is defined using AUTOMATIC_SURFACE_TO_SURFACE, as is the contact in the head–football impact. The static and dynamic friction coefficients between the skin and the concrete are both set to 0.5, while the friction coefficients between the skin and the football are set to 0.78. The complete head impact simulation files are available at https://github.com/Zhang-Yu-Ting/Data-Driven-Brain-Injury-Threshold, accessed on 10 September 2024. Further information about the model can be found in the THUMS documentation on the official THUMS website.

**Table A1:** Material properties of the THUMS, the concrete block, and the football.

Part	LS-DYNA Material Model	Material Properties
Brain	Kelvin–Maxwell viscoelastic	$\begin{array}{l} {\rho =10}^{-9}{\;t/\mathrm{mm}}^{3}\\ K=2.16\;\mathrm{GPa}\\ G_{0}=6\;\mathrm{kPa}\\ G_{\infty }=1.2\;\mathrm{kPa}\\ {\beta =80\;s}^{-1} \end{array}$
Skull	Elastic	$\begin{array}{l} {\rho =10}^{-9}{\;t/\mathrm{mm}}^{3}\\ E=1.09\;\mathrm{GPa}\\ \upsilon =0.22 \end{array}$
CSF/ventricles	Viscoelastic	$\begin{array}{l} {\rho =10}^{-9}{\;t/\mathrm{mm}}^{3}\\ K=2\;\mathrm{GPa}\\ G_{0}=0.5\;\mathrm{kPa}\\ G_{\infty }=0.1\;\mathrm{kPa}\\ {\beta =80\;s}^{-1} \end{array}$
Pia mater/Arachnoid	Elastic	$\begin{array}{l} {\rho =10}^{-9}{\;t/\mathrm{mm}}^{3}\\ E=1.1\;\mathrm{MPa}\\ \upsilon =0.4 \end{array}$
Dura mater	Elastic	$\begin{array}{l} {\rho =10}^{-9}{\;t/\mathrm{mm}}^{3}\\ E=31.5\;\mathrm{MPa}\\ \upsilon =0.45 \end{array}$
Falx/tentorium	Elastic	$\begin{array}{l} \rho =1.13\times 10^{-9}{\;t/\mathrm{mm}}^{3}\\ E=31.5\;\mathrm{MPa}\\ \upsilon =0.45 \end{array}$
Concrete	Elastic	$\begin{array}{l} \rho =2.35\times 10^{-9}{\;t/\mathrm{mm}}^{3}\\ E=1.2\;\mathrm{GPa}\\ \upsilon =0.25 \end{array}$
Football (size 5) (a diameter of 220 mm and an internal pressure of 0.9 bar)	Ogden rubber (Outer panel) (Thicknesses: 2.2 mm)	$\begin{array}{l} \rho =1.17\;t/{\mathrm{mm}}^{3}\\ G=100\;\mathrm{MPa}\\ \upsilon =0.499 \end{array}$
	Ogden rubber (bladder) (Thicknesses: 0.8 mm)	$\begin{array}{l} \rho =0.9\;t/{\mathrm{mm}}^{3}\\ G=100\;\mathrm{MPa}\\ \upsilon =0.499 \end{array}$

## Appendix B

In line with the impact site descriptions in the text, Table A2 lists a total of 50 scenarios, accounting for both impact site and direction, applicable to head–concrete and head–football impacts. In Table A2, α,β,λ represent the direction angles of the impact site’s position vector, and I denotes the impact direction. When I=“0”, it indicates that the impact direction is parallel to the dashed line in Figure 2 of the text, representing a normal impact. When I=“1−x”, it represents an oblique impact. The value x in “1−x” designates different cases of oblique impact.

**Table A2:** Fifty scenarios considering impact site and impact direction.

Scenario	α−β−γ (I)	Scenario	α−β−γ (I)
1	0-90-90 (0)	26	60-45-60 (0)
2	15-90-75 (0)	27	69-30-69 (0)
3	30-90-60 (0)	28	127-60-52 (0)
4	45-90-45 (0)	29	135-45-60 (0)
5	60-90-30 (0)	30	110-30-69 (0)
6	75-90-15 (0)	31	45-90-45 (1-1)
7	90-90-0 (0)	32	45-90-45 (1-2)
8	105-90-15 (0)	33	45-90-45 (1-3)
9	120-90-30 (0)	34	45-90-45 (1-4)
10	135-90-45 (0)	35	90-90-0 (1-1)
11	150-90-60 (0)	36	90-90-0 (1-2)
12	165-90-75 (0)	37	90-90-0 (1-3)
13	180-90-90 (0)	38	90-90-0 (1-4)
14	90-0-90 (0)	39	180-90-90 (1-1)
15	105-15-90 (0)	40	180-90-90 (1-2)
16	120-30-90 (0)	41	180-90-90 (1-3)
17	135-45-90 (0)	42	180-90-90 (1-4)
18	150-60-90 (0)	43	90-0-90 (1-1)
19	165-75-90 (0)	44	90-0-90 (1-2)
20	90-75-15 (0)	45	90-0-90 (1-3)
21	90-60-30 (0)	46	90-0-90 (1-4)
22	90-45-45 (0)	47	60-45-60 (1-1)
23	90-30-60 (0)	48	90-45-45 (1-1)
24	90-15-75 (0)	49	135-45-60 (1-1)
25	52-60-52 (0)	50	135-45-90 (1-1)

## Appendix C

The method for extracting head impact velocity from a backward fall video is shown in Figure A3. The example is taken from a common life scene without any identifiable features, such as people or residences. By referring to the table in the figure, we determined the vertical direction in all frames and calculated the ratio between the image length (2.56 cm) and actual length (75 cm). In the frames immediately before landing, we further determined that the child’s head translation direction was parallel to the line connecting points $h_{1}$, $h_{2}$, and $h_{3}$. Points $p_{2}$ and $p_{1}$ represent the head’s position at the moment of impact and in the previous frame, respectively. The displacement was decomposed into vertical (25.2 cm) and horizontal (13.8 cm) components. Based on the 80 ms time interval between the frames, we calculated the average velocity as the head’s impact speed. The velocities obtained from two videos are presented in Table 1 of the text.
